# Commensal microbiota drive the functional diversification of colon macrophages

**DOI:** 10.1038/s41385-019-0228-3

**Published:** 2019-11-26

**Authors:** Byunghyun Kang, Luigi J. Alvarado, Teayong Kim, Michael L. Lehmann, Hyeseon Cho, Jianping He, Peng Li, Bong-Hyun Kim, Andre Larochelle, Brian L. Kelsall

**Affiliations:** 1 National Institute of Allergy and Infectious Diseases, Lung and Blood Institute, NIH, Bethesda, MD 20892 USA; 2 National Heart, Lung and Blood Institute, NIH, Bethesda, MD 20892 USA; 3 San Diego State University, 5500 Campanile Dr., San Diego, CA 92182 USA; 4 National Institute of Mental Health, NIH, Bethesda, MD 20814 USA; 5 National Laboratory of Cancer Research, NIH, Frederick, MD 21702 USA

## Abstract

Mononuclear phagocytes are a heterogeneous population of leukocytes essential for immune homeostasis that develop tissue-specific functions due to unique transcriptional programs driven by local microenvironmental cues. Single cell RNA sequencing (scRNA-seq) of colonic myeloid cells from specific pathogen free (SPF) and germ-free (GF) C57BL/6 mice revealed extensive heterogeneity of both colon macrophages (MPs) and dendritic cells (DCs). Modeling of developmental pathways combined with inference of gene regulatory networks indicate two major trajectories from common CCR2^+^ precursors resulting in colon MP populations with unique transcription factors and downstream target genes. Compared to SPF mice, GF mice had decreased numbers of total colon MPs, as well as selective proportional decreases of two major CD11c^+^CD206^int^CD121b^+^ and CD11c^−^CD206^hi^CD121b^−^ colon MP populations, whereas DC numbers and proportions were not different. Importantly, these two major colon MP populations were clearly distinct from other colon MP populations regarding their gene expression profile, localization within the lamina propria (LP) and ability to phagocytose macromolecules from the blood. These data uncover the diversity of intestinal myeloid cell populations at the molecular level and highlight the importance of microbiota on the unique developmental as well as anatomical and functional fates of colon MPs.

## Introduction

Local microenvironmental cues are critical to imprint tissue-specific functions of MPs via driving unique enhancer landscapes and transcriptional programs.^1,2^ In the intestine, circulating Ly6c^hi^ monocytes constantly replenish a majority of the MP pool by replacing embryonic precursor-derived MPs around the time of weaning, which is driven largely by the microbiota.^3–6^ Differentiation from Ly6c^hi^MHCII^lo^ blood monocytes to Ly6c^lo^MHCII^hi^ intestinal MPs correlates with dynamic changes of many MP maturation markers, including the upregulation of CD11c, CD64, CX3CR1, F4/80, and MerTK.^7–9^ Additionally, discrete mature MP subsets have been defined with regards to the expression of CD11c both in small intestine and colon in the steady-state.^5,10,11^ Furthermore, CD4 and TIM4 are expressed by a subpopulation of MPs in the small intestine and colon, with TIM4 expressed solely by locally maintained colon MPs,^12^ possibly derived from embryonic or blood precursors early in life.^13^ Finally, CD169^+^ macrophages have also been described in the colon, their differentiation in the steady-state dependent on Vitamin A, and during intestinal inflammation secrete CCL8 to recruit inflammatory monocytes.^14,15^

Resident intestinal MPs are thought to play an essential role in killing invading microbes, clearing dead and dying cells, control of intestinal inflammation, and contributing to wound healing and epithelial repair,^16,17^ and they are highly phagocytic and bactericidal cells that respond to TLR ligands with the production of IL-10, and other anti-inflammatory, but low levels of inflammatory cytokines,^5,8,18–20^ and also have been reported to be important in the expansion or survival of regulatory T cells within the lamina propria through their production of IL-10 during steady state and colitis.^21,22^ Several tissue-specific factors affecting intestinal MP identity and function have been described, including retinoic acid, microbial metabolites, TGFβ, and IL-10, however, other influences on intestinal MP phenotype are largely unknown.^16^ IL-10 and TGFβ appear to affect the expression of unique sets of genes in colon MPs.^9^

During intestinal inflammation in several murine models of inflammatory bowel disease (IBD), blood monocytes become largely CD11c^−^CX3CR1^int^Ly6c^hi^ MHCII^+^ inflammatory/effector monocytes, that are thought to play a major role in chronic colitis and IBD through their production of inflammatory cytokines,^5,8,23^ but may also generate monocyte-derived “DCs”, with the ability to prime T cells, and possibly migrate to the MLNs.^23,24^ CX3CR1^hi^ colon MPs during colitis appear to largely maintain their regulatory phenotype,^23^ however the origin of these regulatory MPs during colitis is still unclear.^5,8,23^

A role for the commensal microbiota has been implicated in colon MP differentiation and/or maintenance in several studies, with lower numbers of both monocyte-derived and tissue-resident long-lived MPs present in germ-free (GF) mice,^5,6,12,25,26^ however, a substantial number of mature colon MPs are still found in adult GF mice.^5,6,12^ In addition, it was recently shown that antibiotic exposure causes intestinal MPs to become hyper-responsive to bacterial exposure, resulting in enhanced cytokine production and long-term enhanced Th1 responses and dysbiosis.^26^ Despite these advances, the precise developmental relationship of the diverse MP populations in the steady-state colon, as well as the influence of commensal bacteria on intestinal MP developmental pathways and functional phenotypes remain unclear. To address these issues, we performed single cell (sc-RNA) analysis of mRNA of MHCII^hi^ colon mononuclear phagocytes from both GF and specific pathogen-free (SPF) mice, together with tissue-staining for surface markers, and functional assays of antigen uptake.

## Results

### Single cell RNA sequencing identifies heterogenous subsets of mouse colon MPs and DCs

**Fig. 1:**
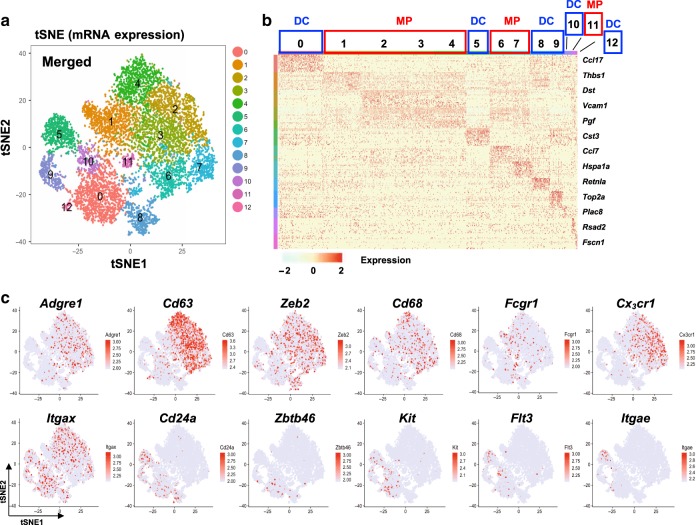
Extensive Heterogeneity of Phagocytes in the Mouse Colon. **a** tSNE plot of 9399 cells from SPF (4681 cells) and GF (4718 cells) colon. Graph-based clustering defined 13 clusters of colon phagocytes. **b** Heatmap of top 30 differentially expressed genes of each cluster from the merged data. The most differentially expressed gene of each cluster is labeled. **c** Feature plots showing macrophage (upper panel) and dendritic cell (lower panel) signature genes.

Single-cell mRNA gene expression profiling^27^ of mature myeloid cells (Lin^−^MHCII^hi^) from the colon of SPF and germ-free GF C57BL/6 mice was performed as outlined in Supplementary Fig. 1a. Graph-based clustering^28^ of gene expression profiles from approximately 5000 individual cells from SPF and GF mice revealed extensive heterogeneity, identifying 13 cell populations (Fig. 1a). Each population was identified by unique sets of differentially expressed genes (DEGs) (Fig. 1b, and Supplementary Table 1): 7 of the clusters were classified as colon MPs, based on known markers, including *Adgre1* (F4/80), *Cd63*, *Cd68*, *Cx3cr1*, and *Zeb2*, essential for MP development^29^ (Fig. 1c), and 6 as DCs, based on marker including *Zbtb46, Kit, Flt3*, and *Itgae* (CD103) expression, and comparison of gene expression for each cluster with the ImmGen database^30^ (Supplementary Fig. 1c). A summary of the colon MP clusters identified and analyzed in this paper, together with their defining mRNA and surface markers can be found in Table 1.

**Table 1:** Summary of colon MP populations.

Cluster	Top5 Cluster Gene Markers	Surface markers evaluated	mRNA for known MP genes^a^	Differentiation Status predicted from monocle2	Specific active regulons^b^	References^c^
1	*Thbs1, Ccr2, Tgm2, Emilin2, Ifi27l2a*	CD11c^−^CD206^−^CCR2^+^	*Ccr2*	Precursor cells (unaffected in GF mice)	*Mxi1*	^3–9,23,26,29^
2	*Cx3cr1, Dst, H2-M2, Dnmt3a, Vcam1*	*n.d*.^d^	*Itgax*	Transional and mature cells (increased in GF mice)	*Nfic, Tcf4, Mef2a, Ddit3, Nle2l2, Mitf, Fosb*	^26^
3	*Apoe, Ms4a7, Cd63, Vcam1, Hpgds*	*n.d*.	*Itgax*	Transitional cells (reduced in GF mice)	*Spic, Nfic, Tcf4, Mef2a, Ddit3,Mitf, Fosb*	^3–12,23,26,29^
4	*Pgf, Mmp13, Dnase1l3, Acp5, H2-M2*	CD11c^+^CD206^int^CCR2^−^CD121b^+^	*Itgax*, *Cd74, IL1r2*, *Cd9, Cd81*	Mature cells (reduced in GF mice)	*Spic, Nifc,Tcf4, Pdrm1,Crem1,Fosb, Mxi1*	^3–12,18,23,26,29^
6	*Ccl7, Ccl2, F13a1, Pf4, Cd163*	CD11c^−^CD206^hi^CCR2^−^CD169^+^	*Mrc1*(hi), *Cd36, CD163,F13a1*	Mature cells (reduced in GF mice)	*Jund, Tcd4, Nfic, Mafk, Atf4, Erg2, Mef2a*	^5,10,11,14,15,18,19,25,29,38,39^
7	*Hspa1a, Hes1, Hspa1b, Ccl4, Jun*	*n.d*.	*Itgax*	Mature cells (unaffected in GF mice)	*Erg1, Erg2, Jun, Irf1, Atf3, Fosb, Mef2a, Nfic*	^3–12,29^
11	*Ifit2, Ifit1, Rsad2, Isg15, Cxcl9*	*n.d*.		*n.d*.	*Stat1, Stat2, Erg2, Irf1, Irf7*	

^a^all cells express *Adgre1,Fcgr1,Cd63,Zeb2,Cd68,Cx3r1; with cluster 1 expressing less Adgre1, Cd68, Cx3cr1* (see Fig. 1c, and Supplementary Tables)

^b^predicted compared to all MP and DC clusters (see Fig. 5f)

^c^primary manuscripts describing related cell types

^d^not determined

### Commensal microbiota globally affects the development and gene expression of colon MPs

**Fig. 2:**
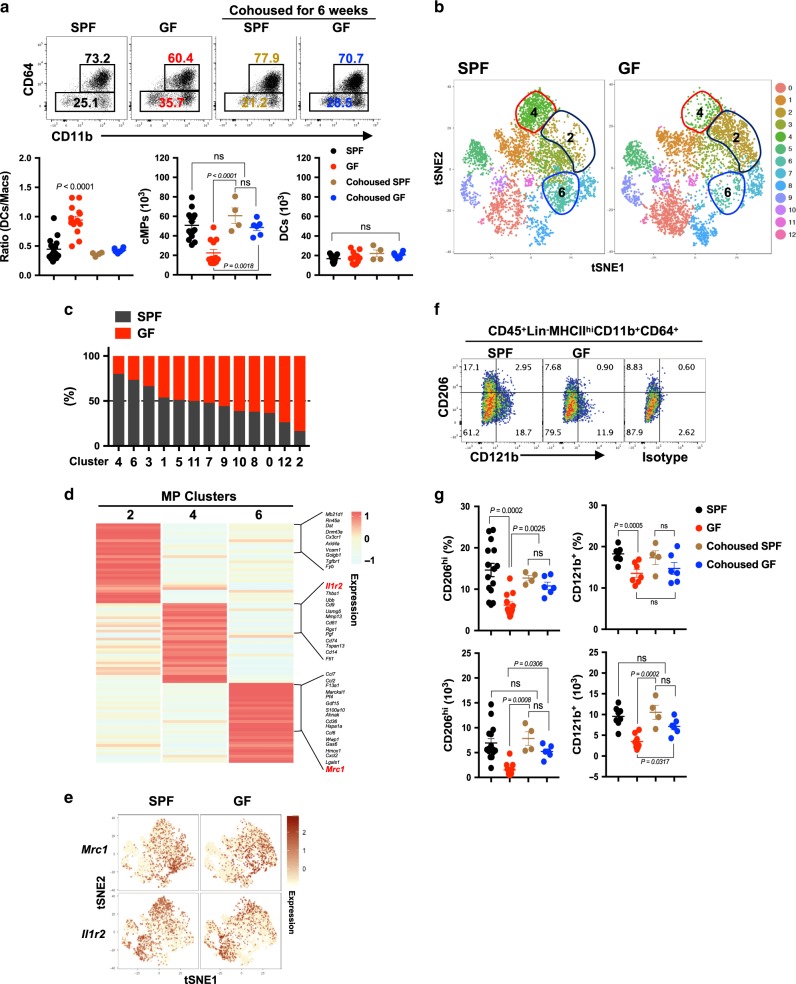
Microbiota Affects Heterogeneous Colon Macrophage Development. **a** Representative flow cytometry data to show the percentage of CD64^+^ MPs and CD64^−^ DCs from SPF, GF and co-housed SPF and GF mice, and quantification of DCs vs MPs ratio and absolute number of MPs and DCs. **b** tSNE plot of SPF and GF phagocytes. Cluster 2, 4, and 6 with highlights. **c** Percentage of SPF or GF cells of the total cells within each cluster from the merged dataset. **d** Heatmap of top40 differentially expressed genes in between cluster 2, 4, and 6. *Il1r2* and *Mrc1* were selected as surface markers for cluster 4 and 6, respectively, and highlighted in red. **e** Feature heatmap of *Mrc1* and *Il1r2* in SPF and GF cells. **f** Representative plot to show the surface staining of CD206 and CD121b (encoded by *Mrc1* and *Il1r2* genes, respectively) on colon macrophages from SPF and GF mice. **g** Summary of percentage and absolute number of either cell type. Data from SPF (*n* = 15), GF (*n* = 12), and co-housed SPF (*n* = 4) and GF (*n* = 6) mice ± SEM from two independent experiments.

We initially compared the total numbers of colon DCs and MPs in GF, SPF mice, and conventionalized GF mice by flow cytometry. GF mice had a decreased total number of colon MPs (CD64^+^) but not DCs (CD64^−^), resulting in a significantly increased ratio of DCs to MPs, and these changes were reversed by co-housing of GF with SPF mice (Fig. 2a), indicating a selective effect of microbiota on colon MPs but not DCs.

Next we determined the influence of the commensal microbiota at the single-cell level, by generating separate tSNE plots of gene expression for SPF and GF mice from the combined dataset (Fig. 2b). We first sought to understand how the microbiota influences the global gene expression of colon MP populations by evaluating the DEGs in all MP populations (Cluster 1, 2, 3, 4, 6, 7, and 11) from SPF compared to GF mice (Supplementary Fig. 2a, b). Colon MPs from SPF mice had increased expression of genes associated with immune defense, antigen presentation, oxidative phosphorylation and gene translation (ribosomal proteins), and decreased expression of genes associated with RNA splicing and chromatin reorganization, suggesting global effects of microbiota on metabolism, gene regulation with epigenetic modifications, host defense, and adaptive immunity.

### Commensal microbiota promotes the development of CD11c^+^CD121b^+^ and CD11c^−^CD206^hi^ MPs

We next evaluated the influence of the commensal microbiota on specific cell clusters (Fig. 2c). A major decrease in the proportions of colon MP clusters 4 and 6 and an increase in cluster 2 were observed in GF compared to SPF mice (Fig. 2b), with less to no effects on clusters 7 and 11. In contrast, the proportion of colon DC clusters 0, 5, 8, 10 in GF mice were modestly increased or unchanged compared to SPF mice, while cluster 12 was more significantly increased. To validate these findings, selective surface markers were identified by transcriptomic analysis for colon MP cluster 4 (*Il1r2*/CD121b) and 6 (*Mrc1*/MMR/CD206) (Fig. 2d, e), while no antibodies to specific surface markers were identified for clusters 2 or 7. Flow cytometric analyses showed lower percentages of both populations in GF compared to SPF mice, and these differences were reversed again by co-housing (Fig. 2f, g).

Next, we evaluated DEGs between cluster 4 (CD11c^+^CD121b^+^) and cluster 6 (CD11c^−^CD206^hi^), which are decreased in GF mice (Supplementary Fig. 3a; Supplementary Table 2). Functional annotation of DEGs revealed organic acid transport, antigen processing and presentation, cell killing, lipid localization and myeloid leukocyte migration as the most significant functions associated with cluster 4, whereas the most significant functions associated with cluster 6 were cellular response to interleukin 1, wound healing, regulation of vasculature, apoptotic cell clearance, and myeloid leukocyte cytokine production. CD121b (*Il1r2*) expressed by cluster 4 is induced by regulatory cytokines, such as IL‐4, IL-13, IL‐10, and controls the proinflammatory functions of IL-1 by inhibiting IL-1β signaling and IL-1α processing.^31^ Cluster 4 also expressed higher levels of TGFβ-dependent genes (Supplementary Fig. 2c) including *Apoe, Rgs1*^32^ *and Thbs1*, which together with *Mmp13* can contribute to TGFβ activation,^33^ as well as the C-type lectin receptors, *Clec7a* (Dectin-1) and *Clec4n* (Dectin-2), suggesting specialized innate immune functions in response to fungi or other pathogens.^31^ In contrast, genes highly expressed in cluster 6 included chemokines (*Ccl2, Ccl6, Ccl7, Ccl8, Pf4*) involved in chemotaxis of monocytes and other leukocytes, scavenger receptors (*Cd163, Cd36* and *Stab1*) associated with M2/wound healing/regulatory macrophages, and genes involved in vascular interactions (*Lyve1*), hemostasis (*F13a*), migration (*Ahnak*) and suppression of proinflammatory gene expression (*Atf3*).^34^

Cluster 2, which was dramatically increased in GF mice, expressed lower levels mRNA for genes associated with the inflammatory response, myeloid leukocyte migration, MyD88-independent TLR4-cascade and stress responses compared to all other colon MP subsets (Supplementary Fig. 3b; Supplementary Table 3). Notable genes that were poorly expressed in cluster 2 included *Nfkbia, Nfkbiz, Ftl1* (ferritin light chain) and *Ubb*, associated with decreased colon MP activation and oxidative stress. In contrast, these cells expressed higher levels of mRNA for TGFβ-dependent genes (Supplementary Fig. 3c), including *Tgfbr1* and *Cx3cr1*.^9,35,36^ Also increased in this subset was *Mb21d1* (cGAS), a gene known to be induced by IFNα/β signaling, which is itself largely driven by a positive feed-forward loop in response to cGAS/STING signaling induced by intracellular DNA from viral infection, DNA damage or leakage from mitochondria.^37^ Together, these results suggest that under GF conditions, colon MP differentiation is diverted to a separate population due to lack of LPS/bacteria signals, with preserved TGFβ, and possibly IFNα/β signaling.

### Unique spatial and functional features of CD11c^+^CD121b^+^ and CD11c^−^CD206^hi^ colon MPs

**Fig. 3:**
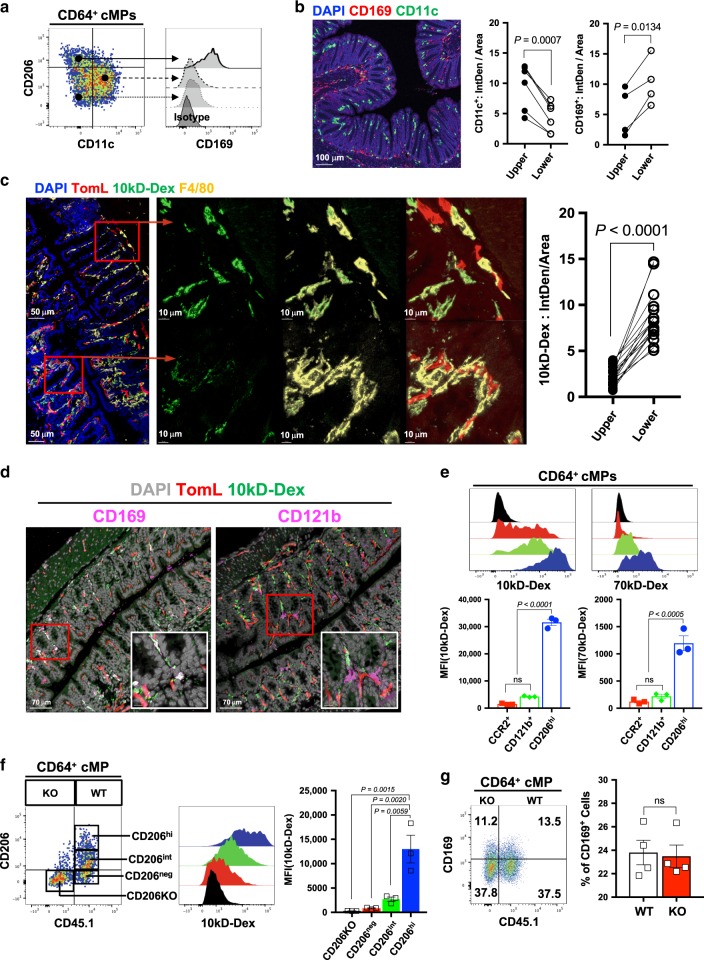
Spatial and functional differences between CD11c^−^CD169^+^(CD206^hi^) and CD11c^+^CD169^−^CD121b^+^(CD206^int^) colon macrophages. **a** Representative histogram of CD169 expression. **b** Representative image of cross-section of CD11c-eYFP reporter colon stained with CD169. scale bar 100 μm. Lamina propria region was separated in half into upper (distal) and lower (proximal) regions (left panel), and integrated density (IntDen) of CD11c (green) and CD169 (red) was quantitated from 6 or 4 sections of two mice (right panel). paired *t*-test. **c** Representative tissue section of C57BL/6 mice intravenously injected with 10kD dextran (green) and tomato lectin (TomL, red) was further stained with anti-F4/80 (yellow) or **d** with CD169 or CD121b (purple). IntDen of 10 kD dextran in the upper and lower parts of lamina propria was summarized in the right panel **c** (17 sections from three mice). paired *t*-test. **e** Representative flow cytometry of phagocytosed 10 or 70 kD dextran by CCR2^+^ (red), CD121b^+^ (green) and CD206^hi^ (blue) subsets of colon MPs. Non-injected mice were used as control (black, top) and the summary of mean-fluorescence intensity (MFI) for dextran of colon MP populations is shown (bottom). **f** Mixed bone-marrow (BM) chimeric mice (CD45.12 wild-type + CD45.2 *Cd206/Mrc1*^*−/−*^ → CD45.1, *n* = 3) were generated and intravenously injected with 10kD dextran. Blood dextran uptake was examined in CD206^hi^ (blue), CD206^int^ (green) and CD206^neg^ (red) colon MPs from wild-type BM and CD206^neg^ colon MPs from *Mrc1*^*−/−*^ BM. The histogram of MFI of dextran intensity (middle panel) and the summary of the intensity (right panel). **g** Representative dotplot of CD169 expression from WT and KO colon MPs from mixed BM chimeras, and the percentage of CD169^+^ MP (*n* = 4).

The distinctively different gene expression patterns between colon MP cluster 4 (CD11c^+^CD121b^+^) and cluster 6 (CD11c^−^CD206^hi^), suggested their differential localization within the colon tissue. To address this possibility, we used CD169 as a more specific marker for cluster 6 (CD206^hi^ colon MP) (Fig. 3a) instead of CD206, a pan-MP marker which is more broadly expressed at lower levels in other colon MPs; and CD11c and CD121b for cluster 4. Interestingly, CD11c^+^ (Fig. 3b) and CD121b^+^ (Fig. 3d) cells localized more distally, closer to the tips of the villi where exposure to apoptotic epithelial cells, and intestinal bacteria and their products would be more prominent. In contrast, the majority of CD169^+^ cells are concentrated closer to the crypt base, as well as within the submucosa^14,38^ (Fig. 3b). Therefore, distinct gene expression profiles between CD11c^+^CD121b^+^ and CD11c^−^CD206^hi^ colon MPs may be explained by their exposure to microenvironments within the tissue, including their proximity to commensal bacteria which is significantly decreased within the crypts.

We next determined the ability of these two colon MP sub-populations to capture systemic antigens, since prior studies had identified CD169^+^ MPs in the spleen and LNs as “gatekeepers” at the blood-LN interface.^38,39^ F4/80^+^cells closely associated with blood vessels avidly took up systemically administered 10kD dextran (Dex) (Fig. 3c), which was further localized to the CD11c^−^CD169^+^CD206^hi^ cell population (Fig. 3d, e), and which was also apparent with 70kD Dex administration. The capacity to capture Dex was solely dependent on the expression of CD206 (Fig. 3f)^40^ and was acquired during colon MP maturation because CCR2^+^ cells, immature monocyte/MPs, were unable to capture 10kD Dex (Supplementary Fig. 4a, b). However, the absence of CD206 had no effect on CD169^+^ colon MP development (Fig. 3g). Surprisingly, in contrast to Dex, all colon MPs were able to take up ovalbumin (Ova) from the blood to varying degrees (Supplementary Fig. 4c–e). These data highlight a spatial, as well as functional separation of the colon MP sub-populations in their ability to capture blood-born antigens, which depends on their associated surface receptors, and likely other, as yet undetermined properties.

### Trajectory analysis suggests developmental bifurcation of colon MPs

**Fig. 4:**
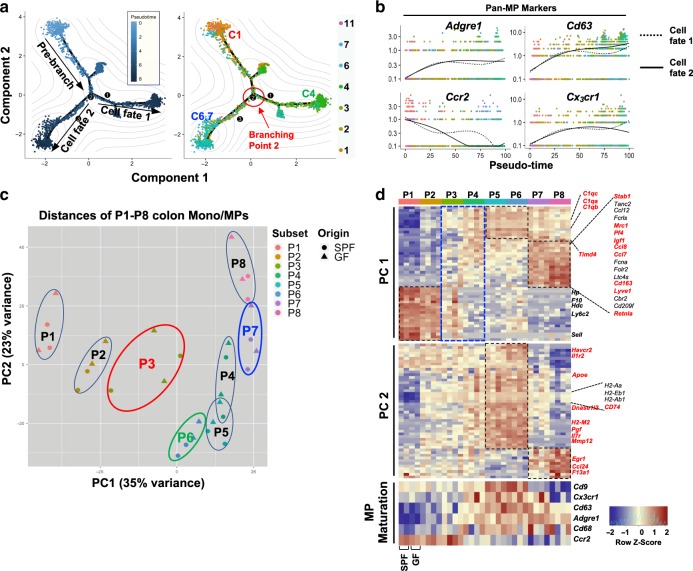
Colon MP diversification from CCR2^+^ common precursors. **a** Developmental trajectory analysis of colon macrophages (cluster 1, 2, 3, 4, 6, 7, 11 from scRNA-seq data) with pseudo-time (left panel) and cell cluster identity (right panel). Cluster 1 (red: *Ccr2*^*+*^), 4 (green: *Il1r2*^*+*^), and 6 (blue: *Mrc1*^*hi*^) and 7 accumulated at pre-branch, cell fate 1 and cell fate 2, respectively. **b** Dynamic change of expression of MP maturation markers (*Adgre1, Cd63, Cx3cr1* and *Ccr2*) along the pseudo-time. **c** Principal component analysis (PCA) of gene expression using bulk RNA-seq of P1–P8 populations sorted from steady state colon defined as in Supplementary Fig. 5a-c. Samples from SPF (•) or GF (▴) mice. P3 (red), P6 (green), and P7 (blue) are the counterparts of cluster 1 (red: *Ccr2*^*+*^), 4 (green: *Il1r2*^*+*^), and 6 (blue: *Mrc1*^*hi*^) from scRNA-seq data, respectively. **d** Heatmap displaying top50 genes of determining the variance of PC1 or PC2, and MP maturation markers. Genes highlighted in red represent the genes that are also highly expressed in the corresponding cell subsets from scRNA-seq data. Subset-specific genes are in black dotted boxes. Blue dotted box highlights the transitional expression pattern of PC1 genes from monocytes to MPs. First two and later two in each subset (P1–P8) are from SPF and GF, respectively.

To explore developmental relationships between colon MP populations, and whether alterations in developmental pathways could account for the changes in colon MPs in GF conditions, we performed developmental trajectory analysis^41–43^ (Fig. 4a). One precursor and two major cell fates were identified with 1 major and 2 minor developmental branch points (BP). When individual colon MP clusters were mapped onto the cell trajectories, cluster 1 was identified as the major precursor with cluster 4 developing in opposite trajectories from clusters 6 and 7 at BP2. A progressive loss of *Ccr2* and an enhanced expression of pan-macrophage markers *Adgre1*, *Cd63*, and *Cx3cr1* over the pseudo-time course, is consistent with development of mature colon MP along both trajectories from *Ccr2*^*+*^ precursors (Fig. 4b).^9^

To support the scRNA-based definition of these divergent cell types, 8 (P1-P8) different sub-types of colon MPs including Ly6c^hi^MHCII^−^ monocytes (P1) and Ly6c^hi^MHCII^+^ intermediate cells (P2) based on the monocyte to macrophage developmental “waterfall”^7,8^ (Supplementary Fig. 5a-c), which are not included in the current scRNA-seq data, were sorted by flow cytometry from SPF and GF colon cells, and subjected to bulk RNA-seq analysis. Principal component analysis (PCA) of bulk RNA-seq data from each subset showed a progressively increasing transcriptional distance between subsets (Fig. 4c): principal component (PC) 1 (*x*-axis) separated P1 and P2, tissue-infiltrated monocytes, from all other mature cells (P4 – P8), while P3 is in an intermediate position. *Hp, F10, Hdc, Ly6c2* and *Sell*, markers of blood monocytes^1,44^ are highly expressed in P1 and P2, and gradually decreased during the transition to P4. In contrast, genes for complementary receptors, scavenger receptors and pan-MP markers (*Cd9, Cx3cr1, Cd63, Adgre1*, and *Cd68*) begin to be expressed during this transition, with a deviation of transcriptional patterns emerging for P5-6 and P7-8 (Fig. 4d, upper and lower panel). In contrast, PC2 is more relevant to explain the distance between mature subsets (P5-6 versus P7-8; *y*-axis). Genes up-regulated in P5 and P6 (Fig. 4d, middle panel) significantly overlap with the genes expressed in cluster 4 by scRNA-seq, and those in P7 and P8 overlap with the genes expressed in cluster 6 by scRNA-seq (Supplementary Table 1), supporting the developmental bifurcation indicated by computational analysis.

Interestingly, P8, an LYVE1-expressing subset of CD206^hi^ colon MPs (Supplementary Fig. 5c–e) that are localized in the submucosa near lymphatic vessels (Supplementary Fig. 6f), showed relatively low levels of mRNA for *Cx3cr1* and genes related to antigen presentation (*H2-Aa, H2-Eb1, H2-Ab2, H2-M2, H2-Oa*, and *Cd74*) compared to other mature subsets, suggesting a differential role for these cells in the colon. In addition, in further direct gene comparison analysis, genes expressed in P3 (CD11c^−^CD206^int^CCR2^+^), P6 (CD11c^+^CD121b^+^CD206^int^) and P7 (CD11c^−^CD121b^−^CD206^hi^) showed significant overlap with the top 20 genes expressed by their counterpart clusters 1, 4, and 6 in the scRNA-seq dataset (Supplementary Fig. 5g, h). Collectively, these data imply that *Ccr2*^*+*^ precursors (cluster 1) mature into colon MPs along two major developmental trajectories, both of which are driven by commensal bacteria.

### Developmental bifurcation of colon MPs is controlled by unique regulons

**Fig. 5:**
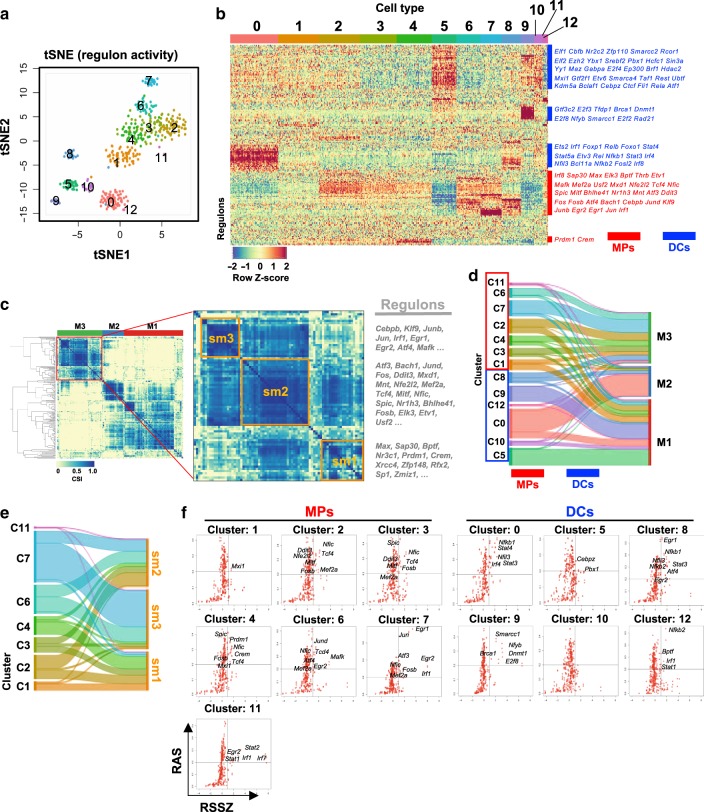
Unique regulon activities dictate colon MP bifurcation. **a** tSNE based on regulon activity scores of avg20. Colors are matched with the mRNA expression pattern-based tSNE plot in Fig. 1a. **b** Heatmap of regulon activity scores. Hierarchically clustered regulons are listed on right side (Red groups: high activity in macrophages; blue groups: high activity in dendritic cells). **c** Three regulon modules (M1–M3) are identified based on regulon connection specificity index (CSI). Close-up view of module M3 identifies sub-modules (sm1 - sm3) and their transcription factor regulons. Sankey plot represents the connectivity between **d** clusters (0–12) of colon phagocytes and regulon modules (M1-M3) or **e** macrophage clusters (1–4, 6, 7, and 11) and regulon sub-modules (sm1–sm3). **f** Plots of RAS and RSSZ (regulon specificity score Z-score) of the regulons in each cluster. Regulons with RAS > 0.2 & RSSZ > 1.0 were considered to be significant and labeled.

The transcriptional state of a cell emerges from an underlying gene regulatory network (GRN) in which a limited number of transcription factors and co-factors regulate each other and their downstream target genes. To gain a better understanding of the GRNs involved in colon MP and DC identity, we used SCENIC (Single-cell regulatory network inference and clustering) to infer the activity of regulons [a transcription factor (TF) together with target genes] for each cell cluster.^45,46^ Visualization of regulon activity scores (RAS) by tSNE plot identified unique patterns of regulon activity for each cell cluster (Fig. 5a). Furthermore, the heatmap of RAS for individual cells identified unique sets of regulons active in colon MP and DC lineages more generally, as well as more specific regulons active in each cell cluster (Fig. 5b).

We next addressed the identity of regulons acting together in the generation of colon MP and DC subsets using an unbiased approach that performs pair-wise comparisons of RAS scores for each cell without regard to its cluster designation and based on the connection specificity index (CSI)^46,47^ (Fig. 5c). Hierarchical clustering identified 3 major regulon modules, each with several submodules. Mapping RAS for regulons within each module to each cell cluster, as visualized in Sanky plots, revealed that M1 regulons were active in both colon MPs and DCs, and M2 and M3 regulons more active in colon DCs and MPs, respectively (Fig. 5d). Since colon MPs are more affected by the microbiome than colon DCs, we further evaluated M3, which has 3 sub-modules (sm1, sm2 and sm3, Fig. 5d, e). Regulons of sm1 are more active in colon MP clusters 1, 2, 3, and 4, sm3 more active in cluster 6 and 7, and sm2 active in all clusters.

Lastly, to define cluster-specific regulons, we analyzed Z-score normalized regulon specificity scores (RSSZ)^47^ together with RAS for each cell cluster (Fig. 5f). We identified the regulons *Crem* and *Prdm1* (sm1) more specific for cluster 4, *Atf4*, *Egr2, Tcd4*, *Mafk* (sm3) and *Jund* (sm2) as more specific for cluster 6, and *Egr1, Egr2, Atf3*, and *Irf1* as more specific for cluster 7, while *Fosb, Nfic, Spic*, and *Mef2a* were more broadly active in most of colon MP clusters. Only *Nfe2l2* distinguished cluster 2, abundant in GF mice, from all other colon MP clusters. To determine how cluster-specific regulons associated with regulon sub-modules control MP differentiation along the trajectory, we examined selected TFs and their target genes that change bidirectionally at BP2 (Supplementary Fig. 6). *Spic, Atf4, Mafk* and *Egr2* contribute to cell fate 1 maturation, *Prdm1* to cell fate 2, and *Jund, and Crem* to both but with proportionately more contribution of *Crem* to cell fate 2, which is also visualized in the RAS heatmap (Fig. 5b). Finally, Gene Ontology (GO) analysis of DEGs for each trajectory from BP2 support bimodal functional maturation (Supplementary Fig. 6). Together, these data identify both general and specific regulons that may contribute to the developmental and functional fates of colon MPs.

## Discussion

Here we present a detailed unbiased single-cell transcriptomic definition of intestinal myeloid cell heterogeneity and the role of microbiota in colon MP development and functions. We demonstrate extensive heterogeneity in colon MPs and DCs, provide data supporting a role for commensal microbiota in the generation of CD11c^+^CD206^int^CD121b^+^ (cluster 4) and CD11c^−^CD206^hi^CD169^+^ (cluster 6) from CD11c^−^CD206^int/lo^CCR2^+^ (cluster 1) colon MPs with relative stemness, as inferred by pseudo-time and GRN analyses, and identify candidate TFs and regulon-networks essential in the course of colon MP maturation. The spatial and functional divergence of colon MPs is consistent with distinctive roles in balancing immune responses to external threats and tissue homeostasis, with CD11c^+^CD206^int^CD121b^+^ colon MPs in the distal LP expressing genes related to immune effector functions, and CD11c^−^CD206^hi^CD169^+^ colon MPs in more proximal LP expressing genes important for cell recruitment, cell scavenging, and tissue regeneration.

We demonstrated that mature macrophages, in particular CD11c^+^CD206^int^CD121b^+^ (cluster 4) and CD11c^−^CD206^hi^CD169^+^ (cluster 6) colon MPs, are significantly reduced in GF mice compared to SPF mice and reversed by recolonization. These findings are seemingly at odds with a previous study reporting that development of CD169^+^ colon MPs is independent of microbiota,^15^ however, this study examined antibiotic-treated, but not GF mice, which are different both immunologically and metabolically.^48,49^ Furthermore, consistent with our results showing that GF mice conventionalized by co-housing with SPF mice for 6 weeks recovered this cell population from the colon, a prior study demonstrated that fecal transplants into GF mice resulted in an increase in CD206-expressing cells in the muscularis layer of colon and small intestine,^25^ indicating a commensal microbiota-dependent mechanism promotes their development.

Exposure of the intestine to bacteria as occurs at birth and weaning, as well as in conventionalized GF mice has significant effects on both the mucosal and systemic immune systems, including the epithelial barrier,^50^ as well as broader systemic effects, including effects on metabolism, at least in part due to dissemination of microbial metabolites.^51^ These changes include the induction of both pro- and anti-inflammatory cytokines in mucosal tissue important for establishing microbial-host mutualism that affects colon MP differentiation/maturation from monocytes.^50^ In this regard, Cluster 4 (CD11c^+^CD121b^+^) colon MP expressed many downstream genes reported to be increased by TGFβR-signaling (Supplementary Fig. 2c). Together with their preferential localization within the colon LP, exposure of these cells to TGFβ produced by epithelial cells, particularly in response to SCFAs from commensal bacteria,^52^ is consistent with their gene expression profile. Furthermore, SCFAs have been shown to have important effects on intestinal MPs, as shown by studies of antibiotic-treated mice.^26^ In contrast, in non-intestinal lymphoid tissues, expression of CD169 by MPs can be driven by type-1 IFNs during viral infections but is constitutively expressed by marginal-zone and subcapsular-sinus MPs that require B cell-produced LTα1β2, as well as RANK, LXR, and CSF-1 signaling.^39^ Other specific signals that may be important for MP differentiation is implied by our analysis of regulons important for these divergent differentiation pathways, and upstream analysis of these pathways is complex, and currently being evaluated.

In contrast to specific signals, our data also implicate more global factors for colon MP differentiation in response to microbiota. One possibility in this regard is that type-2 cytokines (IL4/IL13) and IL-10, as well as prostaglandins and glucocorticoids can induce the expression of CD121b,^31^ the primary marker for this population, but are also implicated in the differentiation of classic “M2” MP, which express high levels of CD206 and CD169 both markers of cluster 6 (CD206^hi^CD169^+^) MPs. Furthermore, when analyzed as a whole, microbiota increased genes in colon MPs related to oxidative phosphorylation, translation and antigen processing and presentation but decreased genes associated with mRNA splicing and transcription (Supplementary Fig. 2). Recently, IL10 and SFCAs (butyrate), were shown to promote the generation of anti-inflammatory^53^ and microbiocidal^54^ MPs by reprogramming metabolism from glycolysis to enhanced oxidative phosphorylation resulting in a reduction in the activity of mTOR, thereby promoting autophagy. Enhanced mitophagy of dysfunctional mitochondria, which is characterized by low membrane potential and a high level of reactive oxygen species, prevented dysregulated activation of NLRP3 inflammasome, and increased xenophagy provided host defense against microbes. Butyrate also enhanced the transcription of antimicrobial peptides through inhibition of histone deacetylase 3. Together with our findings, these studies implicate these, or other factors, result in changes in MP metabolism toward oxidative phosphorylation in response to intestinal microbes, resulting in anti-inflammatory colon MP with increased capacity to kill microbes. It is not clear how these metabolic shifts caused by the presence of microbiota are related to the control of transcription and translation of individual genes, however, microbiota appear to be one of key factors that endow mature colon MPs with tissue-specific functions. Furthermore, microbiota have been shown to have broad physiological effects in the intestine, including nutrient processing and uptake, the differentiation of epithelial cells and maintenance of barrier function, the development of the enteric nervous system, as well as the production angiogenic factors that maintain or induce the development of the enteric vasculature.^55–59^ Therefore, MP development in the colon may be quite complex, likely including both direct and indirect effects of the microbiome that result in specific local niches that allow for the development of unique MP populations.

Here, we focused more on MPs than DCs because the absolute number and the proportion of DCs in the colon of SPF and GF, as well as conventionalized GF mice are not significantly different, with the exception of one population, cluster 12, indicating that colon DCs are less affected by microbiota than colon MPs. The reason why microbiota have these differential effects on DC and MP development is not yet clear. However, consistent with prior studies,^5^ we found that a majority of CD11c^+^ cells in the colon LP co-expressed F4/80, while CD11c^+^ cells lacking F4/80 dominated in isolated lymphoid follicles (ILFs) and colonic patches, indicating that lymphoid follicular structures are major sites where DCs reside in the colon. These findings, together with recent data that unlike ILFs and PPs in the small intestine, the formation of colonic lymphoid follicles are not significantly impaired in GF mice,^60,61^ may explain why colonization with microbiota did not have major effects on the overall number of DCs in the colon.

While the current manuscript focuses on an extensive analysis of colon MP populations, we performed preliminary analysis of DC clusters based on their expression of markers for previously identified populations (data not shown). Based on this analysis, clusters 5 and 12 are most consistent with previously identified DC1 populations [*Itgae* (CD103)^+^, *Itgam* (CD11b)^−^, *Xcr1+, Sirpa- Irf8*^*+*^*, Irf4-*], and clusters 0, 8, and 10 with DC2 populations [*Itgae* (CD103)^−^, *Itgam* (CD11b)^+^, *Xcr1*^*−*^*, Sirpa*^*+*^*, Irf8*^*−*^*, Irf4*^*+*^]. In contrast, Cluster 9 cells express genes indicating active replication (*Mki67, Smc2*, and *Top2a*), and can be separated into two subpopulations that additionally express genes for DC1 (*Irf8, Xcr1*) or DC2 [*Sirpa, Itgae* (CD11b)], suggesting these cells represent actively dividing DCs. Finally, all DC clusters expressed significant levels of *Id2*, a transcription factor reported to be important for DC1 differentiation in the small intestine. Additional analysis will be necessary to further determine unique functions and developmental relationships, including possibly to monocytes (monocyte-derived DCs) of these DC populations.

In addition to DCs, one particular cluster of MPs that was not discussed in detail in the manuscript is cluster 7, which develops along a similar trajectory to cluster 6, and shares a number of regulons, including *Egr2*. The mostly striking gene that is differentially expressed within cluster 7 from other MP clusters is *Hes1*, a member of the helix-loop-helix (HLH) family of transcription factors that suppress gene transcription. In a peritonitis model using *Mx1*-Cre-*Hes1*^fl/fl^ mice, *Hes1* deficiency promoted the expression of *Cxcl1*, resulting in enhanced recruitment of neutrophils into the peritoneum without affecting other cell populations.^62^ Furthermore, LPS-activated BMDMs derived from *Hes1* conditional knockout mice expressed more *Il6* and *Il12b* transcripts compared to control,^63^ implying an immunosuppressive role of *Hes1* in MPs. Additionally, cluster 7 highly expressed *Atf3*,^34^ *Egr1*^64,65^ and *Klf4*,^66^ all shown to be negative regulators of inflammation. Interestingly, the proportions of this cell cluster were not significantly different between SPF and GF mice (Fig. 2c), suggesting these cells are less affected by microbiota than clusters 4 and 6.

Finally, long-living tissue-resident MPs are defined by expression of both CD4 and TIM4^12^ and possibly derived from embryonic or blood precursors early in life,^6,67^ and minimally affected in adult mice by microbiota.^12^ Cluster 7 minimally expressed *Cd4* and *Timd4* (TIM4), as did clusters 2, 3, 4, and 6 and 7, but neither was expressed by Cluster 1 (*Ccr2*^+^) (Supplementary Fig. 7a, b). It is possible that the low transcript coverage of scRNA-seq was unable to provide an accurate profile of cells expressing low levels of *Cd4* and *Timd4*. However, the expression of *Timd4* was consistently detected only in mature (P4-P8) not in immature (P1-P3) monocyte/MPs from our bulk RNA-seq data (Fig. 4d) and, moreover, flow cytometry showed that multiple populations based on the expression of CD206 and CD11c exist within TIM4^−^CD4^−^, TIM4^−^CD4^+^ and TIM4^+^CD4^+^ cells of CD64^+^ colon MPs (Supplementary Fig. 7c). Therefore, the most likely explanation for our lack of identification of these long-lived TIM4^+^CD4^+^ cells as an independent cluster is that they indeed represent multiple MP populations, potentially affected by environmental cues similar to short-lived blood monocyte-derived MPs, even though they are long-lived cells. Indeed, tissue-specific signals are key factors that govern MP identity and function through effects on enhancer landscapes.^1^

Collectively, we believe that the findings presented in this manuscript form the basis for future studies on how tissue myeloid cell development is influenced either directly or indirectly by signals from endogenous microbiota in the intestine, as well as highlight a previously underappreciated interface between the systemic and mucosal immune systems.

## Materials and methods

### Mice

C57BL/6 mice were purchased from Taconic and used at the age of 7–10 weeks. GF C57BL/6 mice were bred in the NIAID Gnotobiotic Facility or obtained from the Gnotobiotic Core, UNC School of Medicine. All mice were maintained in the NIAID Microbiome Program Gnotobiotic Animal Facility and screened for bacterial contamination prior to use. C57BL/6-[Tg]CD11c:EYFP (000307) and B6.129(Cg)-*Ccr2*^tm2.1Ifc/J^(JAX 017586) mice were purchased from Taconic and the Jackson laboratory, respectively. To obtain CD11c^eYFP/+^CCR2^mRFP/+^ mice, B6.129(Cg)-*Ccr2*^tm2.1Ifc/J^ mice were bred with C57BL/6-[Tg]CD11c:EYFP. B6.SJL-*Ptprc*^*a*^*Pepc*^*b*^/BoyJ (JAX 002014) mice were obtained from the Jackson Laboratory. CD45.12 mice were obtained by crossing CD45.1 (JAX 002014) mice with CD45.2 (C57BL/6, Taconic) mice. *Cd206*/*Mrc1*^*−/−*^ mice were generously provided by Dr. Sanghun Lee from David Sacks’s laboratory, NIAID, NIH. All mice were maintained at an American Association for the Accreditation of Laboratory Animal Care–accredited animal facility at the NIAID and housed in accordance with the procedures outlined in the Guide for the Care and Use of Laboratory Animals under an animal study proposal approved by the NIAID Animal Care and Use Committee.

### Isolation and flow cytometric analysis of colon phagocytes

After removing extra-intestinal fat tissue and blood vessels, colons were flushed of their luminal content with HBSS, opened longitudinally, cut into 2-cm pieces, and incubated with HBSS containing 0.015% DTT (15 min, 37 °C in a shaking water bath). Epithelial cells and mucus were removed by extensive washes in HBSS, 5% FCS, and 25 mM Hepes. Colons were cut into small pieces up and digested in complete Iscove’s media containing 167 µg/ml Liberase TL and 30 µg/ml DNase I (Roche) at 37 °C in a shaking water bath for 60 min. The digested cell suspension was then passed through 100- and 40-µm cell strainers and resuspended in FACs buffer (5% FBS, 0.1% NaN3 sodium azide) or Sorting buffer (5% FBS, 1 mM EDTA) depending on the next procedure. For FACS analysis, cells were stained with Fc block/anti-CD16/32 (2.4G2) and live/dead fixable blue dead cell stain kit (Thermo Fisher) according to the manufacturer’s instruction. Further staining of surface targets was done with the following antibodies: α-CD45.2 (104), CD11c (N418), MHC-II (AF6-120.1), F4/80 (BM8), CD64 (X54-5/7.1), CD11b (M1/70), CD45.1 (A20), CD121b (4E2), CD169 (3D6.112), CD206 (C068C2), CD9 (MZ3), CCR2 (SA203G11), LYVE-1(223322), Ly6c (AL-21), B cell and T cell lineages (referred hereafter as Lin, CD19 (RA3-6B2), TCR-β (H57-597), and TCR-γδ (eBioGL3)), and corresponding isotype controls. Samples were briefly fixed with Fixation/Permeabilization Solution kit (BD Biosciences) for 10 min at 4 C. In case of FACs cell sorting, samples were incubated only with Fc block/anti-CD16/32 without live/dead dye, prior to surface staining. Cells were pre-gated on singlet-CD45^+^ hematopoietic cells and then, Lin^−^MHCII^hi^ cells. Whole cells except for F4/80^−^CD11c^−^ populations from Lin^−^MHCII^hi^ cells were sorted for scRNA seq experiments (Supplementary Fig. 1a, b). Ly6c^hi^MHCII^−^ and Ly6c^hi^MHCII^+^ cells were gated from CD45^+^CD11b^+^ Lin^−^CD64^int/+^ for sorting of P1 and P2 subsets (Supplementary Fig. 5a, b). For the purpose of P3-P8 subset sorting or analysis, cells were pre-gated on live-CD45^+^ cells, and then, whole cells except for CD11b^−^CD11c^−^ populations of Lin^−^MHCII^hi^ cells (referred hereafter as APC gate). CD64^+^CD11b^+^ (MPs) or CD64^−^ (DCs) were further analyzed using anti-CD11c, CD206, CCR2, CD9, CD121b, CD169, and LYVE1 antibodies (Supplementary Fig. 5a, c). Data were collected using BD^TM^ LSRII (BD Biosciences) and the obtained data were processed with FlowJo software (Tree Star Inc.).

### Microbiota reconstitution

Germ-free C57BL/6 mice were transferred out of the germ-free isolators and co-housed with SPF C57BL/6 mice purchased from Taconic in at a ratio of 2:3 (SPF:GF) for 6 weeks. This experiment was repeated twice.

### Bulk RNA-seq sample preparation and analysis

Single-cell suspension of colon LP cells was isolated from pooled colons of 3–5 SPF or GF mice and stained (Supplementary Fig. 5a–c) for flow cytometry cell sorting. Processes of cDNA synthesis and sequencing library generation were performed using SMART-Seq v4 Ultra Low Input RNA Kit (Takara), following the manufacturer’s protocol with least modification. 10X Reaction buffer was prepared by mixing 19 μl of 10X lysis buffer and 1 μl of RNase inhibitor provided by the kit. One microliter of 10X Reaction buffer was mixed with 9.5 μl of RNase-free water and put into a 96-well PCR plate, and the plate was always kept on ice throughout the experiment. 100 cells of each subset of colon monocyte/macrophages (P1-P8) were directly sorted into individual wells of reaction buffer-containing in the 96-well PCR plate. First-strand cDNA synthesis and PCR amplification were carried out according to the manufacturer’s recommendations. Tagmentation and indexing of the amplified cDNAs were performed using the Nextera XT library preparation kit (Illumina) with multiplexing primers, according to manufacturer’s protocol. Library fragment-size distribution was assessed using the Bioanalyzer 2100 and the DNA high-sensitivity chip (Agilent Technologies). Quantification of libraries was performed using Qubit 2.0 (ThermoFisher) and Kapa DNA Quantification Kit (Kapa biosystems) before sequencing. Eight subsets from SPF and GF colons were collected twice independently (total 32 samples). Sixteen samples per lane were multiplexed in NextSeq500, single-end 75 cycle × 1. Downstream analysis of demultiplexed fastq files was executed using Partek Flow (Partek): trimming adaptor sequences (13 bp from 5’ and 3 bp from 3′ were trimmed, leaving 57~58 bp with higher than Q30 of quality score in average), alignment to the reference genome (mm10), and quantification to annotation model (mm10_refseq_v86_18_08_01_v2). STAR aligner was used for sequence alignment and the genomic alignments that map uniquely to the set of known Refseq were used as raw input. TPM normalized count matrix was further visualized using gplots 3.0.1, R package, and the function heatmap.2() with the parameters ‘scale = row’, Rowv = FALSE, Colv = “NA” and dendrogram = c(“none”) (per row z-score transformed log (normalized expression), royalblue1-grey0-yellow). For PCAplot and heatmap in Fig. 4b, c, DESeq2 was used: un-normalized transcript count matrix was used as input, low abundant features were removed by strict pre-filtering, DESeq2 data object from the matrix of counts and the metadata table was constructed and further processed with variance stabilizing transformation. PCA was performed by *prcomp* function with default setting, the list of genes highly responsible for PC1 or PC2 variance were extracted by pca$rotation[,“PCx”] and the absolute high top50 genes in either PC1 or PC2 were selected.

### scRNA-seq using dropseq

Single cell suspensions of colon LP cells were prepared from mouse colons of 10–15 pooled 7–10 weeks-old SPF or GF mice and stained as in Supplementary Fig. 1b. Colon phagocytes were sorted using a FACs Aria (BD Biosciences) at NIAID core and immediately moved to single-cell RNA (scRNA) library preparation. scRNA libraries were prepared using Drop-seq method.^27^ Briefly, using a PDMS microfluidic device (FlowJem), single cells were encapsulated with barcoded microparticles (barcoded bead SeqB from Chemgenes) into droplets containing lysis buffer. The flow rates of partitioning oil, single-cell suspension and barcoded microparticles were 15; 4; and 4 mL/h, respectively. Following single-cell encapsulation, droplet breakage, reverse transcription, exonuclease I treatment and PCR amplification were all performed following the standard Drop-seq protocol.^27^ The amplified cDNA was quantified on Qubit 2.0 and BioAnalyzer 2100 with DNA high-sensitivity chip (Agilent) and then fragmented and amplified using the Nextera XT DNA sample prep kit (Illumina). Final libraries were again quantified on Qubit 2.0 and assessed for fragment size and quality using the BioAnalyzer 2100 with a DNA high-sensitivity chip (Agilent). The libraries were sequenced on the Illumina NextSeq 500, using a High Output Kit v2 with 20 bp of Read1 using a custom primer (5′GCCTGTCCGCGGAAGCAGTGGT ATCAACGCAGAGTAC 3′), 60 bp of Read2, and 8 bp of index read1.

### scRNA-seq data analysis using Drop-seq Tools

Read alignment and generation of digital gene data from raw sequence data were performed using STAR v2.5.4a with default settings and drop-seq-tools-1.12 in NIH Helix/Biowulf High Performing Computation (HPC) system. Raw read1- and read2-fastq files were first converted and merged into uBam files. From read 1, 5′ bases 1–12 and bases 13–20 with equal or higher than 10 quality score were tagged as cell barcode and unique molecular identifier (UMI), respectively, and then filtered for sequence alignment. Adapter sequence and poly-A tail sequence contamination were removed before sequence alignment. The file was converted back into fastq file, which was sequentially used as input for STAR aligner. Data were aligned to the *Mus musculus* (mm10) genome and annotated using USCS mm10 gene annotation. The tagged cell barcode and UMI information was mapped again into aligned/annotated sequences, followed by bead synthesis error correction and digital gene expression extraction (DGE). Only cells which showed at least 5000 reads detection were included by setting NUM_CORE_BARCODES = 5000 in DGE extraction step. The extracted cells-by-genes digital gene expression matrix was further analyzed using Seurat (https://github.com/satijalab/seurat or https://satijalab.org/seurat).^28^

### Clustering and visualization using seurat

To generate merged dataset of colon phagocytes of SPF and GF, each matrix was first loaded and used to establish individual Seurat objects in which only cells to express at least 200 genes and only genes to be detected in at least three cells were included. The two objects were merged using MergeSeurat function and the cells with lower than 200 or more than 2500 genes detected were filtered out, finally leaving 4681 cells of SPF and 4718 cells of GF. The merged object was log-normalized to scale.factor = 10,000, used to determine variable genes with low, high cutoff of x (0.0125, 3) and y.cutoff (0.5), resulting in 1688 variable genes, and regressed to nUMI. Then, principal component analysis (PCA) over the list of variable genes was performed. The first 11 PCs selected based on the inflation point of PCElbow and JackStraw plot were used for both clustering and t-stochastic neighboring embedding (t-SNE) for visualization. We performed FindClusters within Seurat with a resolution of 1.2 and RunTSNE to identify 13 clusters of colon phagocytes. Finding positive cluster markers for every cluster compared to all remaining clusters or differentially expressed genes (DEGs) of between specified clusters were calculated using “FindAllMarkers” or “FindMarker” functions, respectively, by Wilcoxon rank sum test (default). To identify a cell type of each cluster, we used Immunological Genome database (ImmGen, https://www.immgen.org)^30^ based on the top 50 genes of each cluster. The expression pattern of a list of gene set was tested on ‘MyGeneset’ menu with the selection of the indicated populations of Microarray V1 database on the figure and the results were displayed as heatmap (Supplementary Fig. 1a). To maximize the visualization of DEGs in between clusters, we used AverageExpression function within Seurat (Fig. 2d, Supplementary Figs. 2b, 3c, and 5e).

### Gene network and functional annotation

DEGs were calculated in between SPF and GF macrophage and the list of the genes were used as an input of string (https://string-db.org/) to extract the gene-gene interaction information, which were subsequently put into Cytoscape 3.7.1 to build a gene network and to further analyze. The gene network was visualized in prefuse_force_directed_layout by combined_score and grouped by Reactome cluster plug-in into five sub-modules (>35 genes), whose genes were again used as an input to string to find out functional profiles (GO and KEGG). For functional profiling of gene sets in other section of this paper, clusterProfiler^68^ or gProfiler R packages were used.

### Pseudo-time trajectory analysis using Monocle2

To analyze a potential trajectory of macrophage subsets, we decided to use Monocle2 package.^42,43^ The identified macrophage datasets (cluster 1, 2, 3, 4, 6, 7, and 11) were separated from total merged data. To directly inherit all the features determined by Seurat into Monocle2, we followed next steps: filter out cells with low number of genes detected (only cells with nGene > 500 remained); Raw data from the filtered Seurat object were used as input of expression matrix; cell.id (cell barcode), cell.ident (Seurat cluster) and orig.ident (SPF or GF) were inherited and reconstructed as phenoData; gene_short_name (matrix row names) and total reads of each gene (row sum of matrix) were also exported from the macrophage Seurat object and reconstructed as featureData. These components (expression matrix, phenoData and featureData) inherited from the filtered Seurat object were used as input to generate new Monocle object. Sizefactors, dispersions and total mRNA counts were calculated and stored in the object. Only genes detected higher than threshold (0.1) and cells in which total mRNA is more than 2280 or <596 genes were remained. Dispersion table of expressed genes was generated, and 839 ordering genes were selected from the table by sub-setting with the parameters of mean_expression > 0.11 and dispersion_empirical > 1 * dispersion_fit. The dimensionality of the data was reduced to two dimensions with log normalization and DDRTree method, and then, order of cells was determined. The bifurcation of gene expression along two branches centered around the branching point 2 was tested with default setting (Fig. S3E). Genes were hierarchically clustered into four clusters by ward.D2 method.

### Inference of regulons and their activities using SCENIC

To construct ﻿GRNs, we used a modified version of the SCENIC (Single-Cell rEgulatory Network Inference).^45^ We pooled data from every 20 cells randomly selected within each cluster and then applied SCENIC to the average gene expression profile of the pooled data.^46^ This simple modification, called Avg20 as in Suo et al., effectively increases the data quality as well as reduces the computational burden in the downstream analyses. SCENIC calculates RAS in each single cell by summing up the area under the recovery curve. ﻿To quantify the cell-type specificity of a regulon, we calculated Regulon Specificity Score (RSS) and ﻿Jensen-Shannon Divergence (JSD) derived entropy-based measure as previously defined.^69^ ﻿Regulon modules were identified based on the Connection Specificity Index (CSI),^47^ which is based on ﻿Pearson correlation coefficient (PCC) of activity scores to identify the associated regulons. Sankey plots have been generated using pySankey package (version 0.0.1) under Python 3.7.1. The connections between levels (all cell clusters vs motif group M1-M3 or MP clusters vs motif sub-group sm1-3) were determined by the number of cells in each cluster with the motif group having AUCZ above the cutoff of 2.0.

### Dextran and ovalbumin uptake in vivo assay and imaging

Fluorescence-conjugated 10kD or 70kD Dextran, or ovalbumin (ThermoFisher) were used for the histochemical evaluation of vascular permeability and intake activity of perivascular cells. 30 min. prior to harvest, isoflurane-anesthetized C57BL/6, CD11c-eYFP or CD11c-eYFP-CCR2-mRFP mice were intravenously injected with 0.1 ml of 5 mg/ml or 1 mg/ml solution of dextran or ovalbumin, respectively. 5 min. prior to harvest, mice were injected i.v. with 0.1 ml DyLight 594-labeled TomL, tomato lectin (Vector Labs). For FACs analysis, mice were sacrificed, and colon tissues were collected for immune cell isolation as described below. Deeply anesthetized mice were perfused with saline followed by ice-cold 4% PFA. Colons were removed, post-fixed for 2 h at RT, followed by 30% sucrose PBS for 24 h and embedded in OCT compound (Tissue-Tek) for freezing. Frozen tissue was sliced into 10-μm-thick sections and kept on −80 °C. Slide was dried at RT sufficiently, mark the barrier around the tissue using hydrophobic barrier pen (Super^HT^) and then, rehydrated, blocked with 10% rabbit or goat serum in 0.1% Tween-20/PBS (PBST), depending on the isotype of 2nd antibodies used, at room temperature (RT) for 1 h. Antigen retrieval was performed using antigen retrieval reagent (Dako) before serum blocking, if required. Staining of pure-form or fluorescence-conjugated primary antibodies was performed by 1:100 or 1:200 ratio in 1% rabbit or goat serum in 0.1% PBST for 24 h at 4 C followed by three rounds of strict washing with 0.1% PBST, each for at least 30 min. Samples were then treated with fluorescence-conjugated 2nd antibodies in 1:1000 ratio in 1% proper serum in 0.1% PBST for 1 h at RT. Nuclei were stained with 4′,6-diamidin-2-fenilindolo (DAPI). These are the antibodies used for imaging: CD169-Alexa647 (clone 3D6.112, Biolegend), LYVE-1-AF700 (clone ALY7, Novus Biologicals), CD121b (Goat IgG polyclonal, Invitrogen), F4/80 (clone CI:A3-1, Abcam), Goat anti-rat IgG-Alexa647 and Rabbit anti-goat IgG-Alexa568 (Invitrogen, Molecular Probes). Images were acquired using a Leica TCS SP5/SP8 microscope (Leica Microsystems, Mannheim, Germany) using a X20 or X40 objective, zoom X1 and processed with Imaris software 9.0.0 (Bitplane AG, Zurich Switzerland). For the quantification of fluorescence intensity from acquired images, Fiji (image J) was used.

### Bone-marrow chimeric mice

B6.SJL-*Ptprc*^*a*^*Pepc*^*b*^/BoyJ (CD45.1) mice at 8–10 weeks of age were irradiated with two split doses of 450 cGy from a 137Cs source with a 4-h interval, followed by BM cell transfer within 24 h of the second irradiation. BM cells was flushed from the tibias and femurs of CD45.12 (wild type) and *Mrc1*^−/−^ mice. A single-cell suspension of BM cells was suspended in ACK lysis buffer to remove red blood cells, filtered through 40-µm cell strainers and incubated for 5 min. at RT and washed thoroughly with 10% FBS-containing PBS twice. Cell number was counted using Cellometer auto 1000 (Nexcelom). Mixed BM cells (3.0 × 10^6^) at a ratio of 1:1 of wild-type and knockout were prepared in a volume of 200 μl of PBS and then, injected intravenously into the lateral tail vein of the irradiated recipients. At 8-9 weeks of post transplantation, the chimeras were subjected to Dextran uptake in vivo assay and analyzed by flow cytometry.

### Statistics analysis

Significant differences in the number of genes detected were analyzed with Wilcoxon matched-pairs rank sum test, meeting distribution assumption with statistical significance accepted when *p* < 0.05. Data generated from flow cytometry were analyzed using GraphPad Prism software (version 7; GraphPad Software, La Jolla, CA) statistically. A dot in graphs indicates percentage or absolute number of cells from individual organism and an error-bar represents the mean values ± SEM. Data comparison was done using an unpaired *t* test if not indicated, and *p* value < 0.05 was considered significant.

## Supplementary information

Supplementary Figures

Supplementary Table 1

Supplementary Table 2

Supplementary Table 3

## Data Availability

The data discussed in this publication have been deposited in NCBI’s Gene Expression Omnibus,^70^ and are accessible through GEO Series accession number GSE137927 (https://www.ncbi.nlm.nih.gov/geo/query/acc.cgi?acc=GSE137927).

## Code availability

R/Python scripts implementing the analysis and figures can be obtained upon request.
