# Supplementary material for: Commensal microbiota drive the functional diversification of colon macrophages
Source: Mucosal Immunol. 2019 Nov 26;13(2):216–29. doi: 10.1038/s41385-019-0228-3 (PMC7039809; doi:10.1038/s41385-019-0228-3)

Supplementary Figure Legends

Supplementary Fig. 1. Identification of cell clusters, and cell types within each cluster.

(a) Brief summary of experiments and data analysis scheme and **(b)** gating strategy for FACs sorting. (**c)** Identification of cell type using reference dataset. Heatmap shows the pattern of top50 differentially expressed genes from each cluster of scRNA-seq within ImmGen database. NKT: natural killer T cells, NK: natural killer, DCs: dendritic cells, MPs: macrophages, Mono: monocytes, Gran: granulocytes.

Supplementary Fig. 2. Differentially expressed genes (DEGs) between SPF and GF colon MPs. **(a)** Network of genes differentially expressed between SPF and GF colon MPs regardless of clusters. Genes, which are increased (red) or decreased (gray) by the presence of microbiota, are sub-grouped into 5 modules with distinct gene functional profiles. **(b)** Heatmap displays the expression pattern of the 5 gene modules defined from the gene network analysis within major SPF and GF colon MP clusters.

Supplementary Fig. 3. DEGs between clusters and the gene functional annotation.

(a, b) Volcanoplots on the left display **a,** DEGs of C2 versus all other MPs (C1, C3, C4, C6, C7 and C11) and **b,** DEGs of C6 versus C4. Genes in red (logFoldChange > 0.5, adj. $p < 0.05$ for **a**; logFoldChange > 0.6, adj. $p < 0.05$ for **b**) of volcanoplots are further used in the heatmap (middle) for visualization and the table of functional annotation on the right. **(c)** Expression pattern of a set of genes reported to be down-regulated in colon MPs from CD11c-Cre-*Tgfbr1*$^{fl/fl}$ mice compared

with *Tgfbr1*$^{fl/fl}$ mice during the transition from monocyte to mature MPs is examined throughout the colon MP clusters identified from the scRNA-seq.

Supplementary Fig. 4. Capacity to take up blood-born antigens is acquired during local MP maturation. C57BL/6, CD11c-eYFP or CD11c-eYFP-CCR2-mRFP double reporter mice were injected intravenously with 10kD dextran or fluorescence-conjugated ovalbumin (Ova) and sacrificed in 30 min. **(a)** Summary of median fluorescence intensity (MFI) of dextran taken up by P1-P3 colon monocytes/macrophages. Representative result from two independent experiments. **(b)** Confocal images of colon from CD11c-eYFP-CCR2-mRFP double reporter mice intravenously injected with 10kD dextran. The selected areas display CD11c^{+}, CCR2^{+} or CD11c^{+}CCR2^{+} double positive cells from the lamina propria. Scale bar, 50mm and 7 mm. **(c)** Representative confocal images of CD169^{+} and CD11c^{+} cells in lamina propria. C57BL/6 (left panel) and CD11c-eYFP (right panel) mice intravenously injected with Ova. **(d)** Summary of median fluorescence intensity (MFI) of dextran taken up by P1-P3 monocytes/macrophages. **(e)** Representative summary of MFI of engulfed ovalbumin from 3 colon MP subsets (out of 3 independent experiments).

Supplementary Fig. 5. Gating strategy and bulk RNA sequencing of colon MPs confirms scRNA-seq results for clusters 1, 4, and 6. (a) Gating strategy of colon MPs (CD45^{+}Lin^{-}MHCII^{hi}CD11b^{+}CD64^{+}), DCs (CD45^{+}Lin^{-}MHCII^{hi}CD11b^{+}CD64^{-}) and (**b-c)** for P1-P8 subsets of colon monocyte/macrophages. (**d-e)** Featureplot and dotplot displaying the expression of *Mrc1* and *Lyve1*. The dot size and color represent the percentage of target gene-expressing cells and the scaled expression level of the gene, respectively. **(f)** Localization of LYVE1-positive colon MP.

Longitudinal section of colon on the upper left panel (scale bar, 100μm) and close-up view of the selected area (red-boxed) on the upper right and lower panels (scale bar, 15μm). LYVE1 (cyan), F4/80 (yellow), and TomL (blood vessels,red). White arrows indicate LYVE1^{+}F4/80^{+} cells. **(g)** Top20 DEGs (cluster markers displayed in averaged expression value) of clusters 1, 4, and 6 from scRNA-seq data are displayed as heatmap and **(h)** the expression of the same genes (top20 cluster markers of cluster 1, 4 and 6) is tested in the results of bulk RNA-seq from CD11c^{-}CD9$^{lo/neg}$CD206^{int}CCR2^{+} (P3: red), CD11c^{+}CD9^{hi}CD206^{int}CD121b^{+} (P6: green) and CD11c^{-}CD9^{int}CD206^{hi}LYVE1^{-} (P7: blue) cells of SPF and GF colon. Gene names are listed on the right side of the graph and the top color bar of each subset is matched with the highlights in PCA plot of main figure 4c.

Supplementary Fig. 6. Gene expression and regulon activity at branch-point 2. Expression of target genes of the selected regulons (*Crem* and *Prdm1* (sm1), *Jund* and *Spic* (sm2), and *Atf4, Egr2* and *Mafk* (sm3)) at branching point 2 of trajectory. Based on the pattern of expression, genes were divided into 4 modules in the heatmap (middle). Sankey plot on the far left displays how each transcription factor is connected to and potentially regulates the gene modules which fluctuate along the pseudo-time into two different branches. The list of genes in each module and their functional GO terms (right panel) are shown on the far right: gene module 1 and 4 is increased in cell fate 1 (red) whereas gene module 2 and 3 is increased in cell fate 2 (blue).

Supplementary Fig. 7. Long-lived TIM4^{+}CD4^{+} cells are of multiple MP populations.

(a-b) Featureplot and dotplot showing the scaled expression of *Cd4* (CD4) and *Timd4* (TIM4). Featureplot shows the location of positive cells within tSNE coordinate, and in the dotplot, the dot

size and color represent the percentage of positive cells within each cluster and the level of gene expression, respectively. **(c)** Gating strategy of colon MPs and DCs. Surface expression of CD206 and CD11c was evaluated in TIM4$^-$CD4$^-$, TIM4$^-$CD4$^+$ and TIM4$^+$CD4$^+$ sub-populations of colon MPs (CD64$^+$) by flow cytometry.

Supplementary Fig. 1

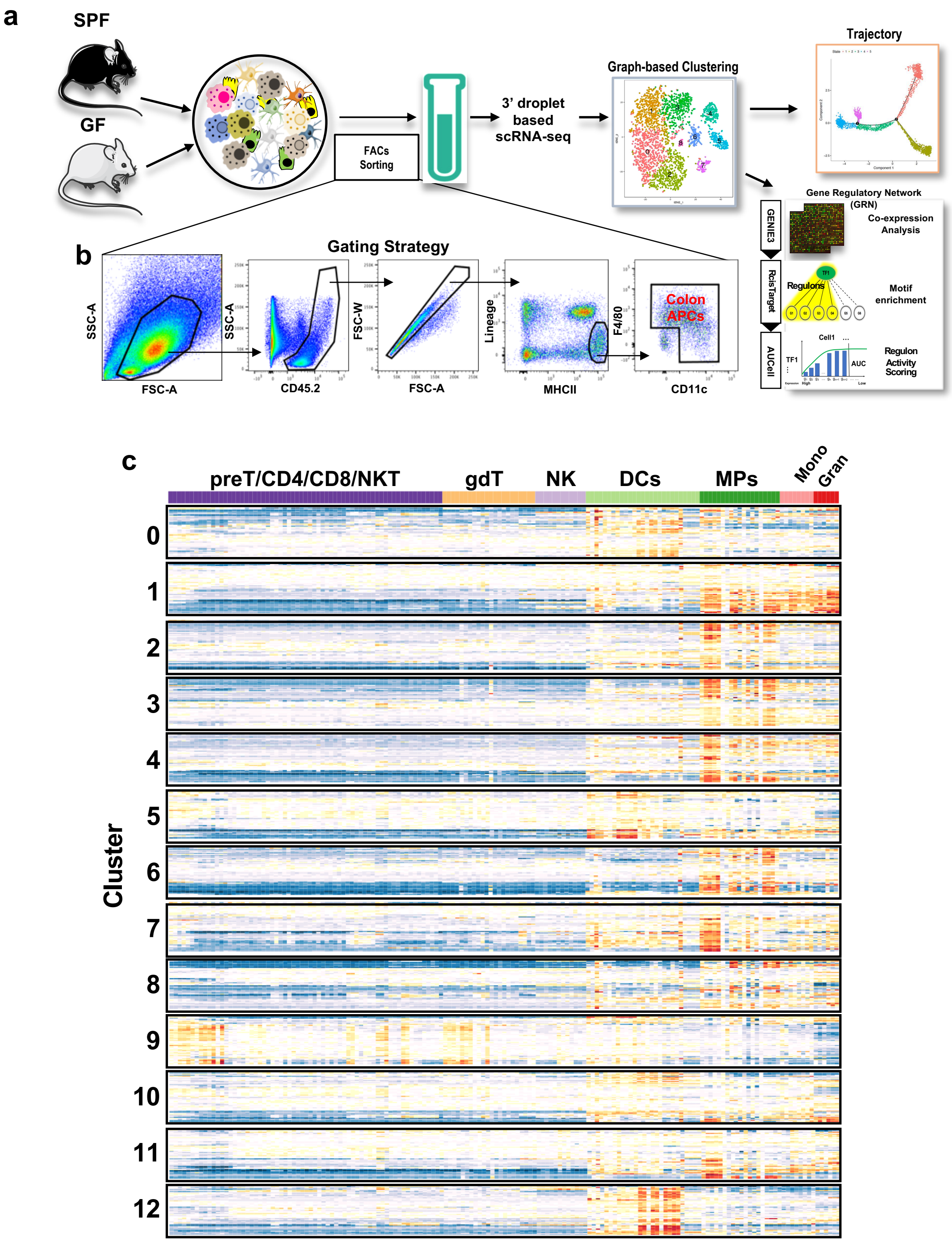

Supplementary Fig. 2

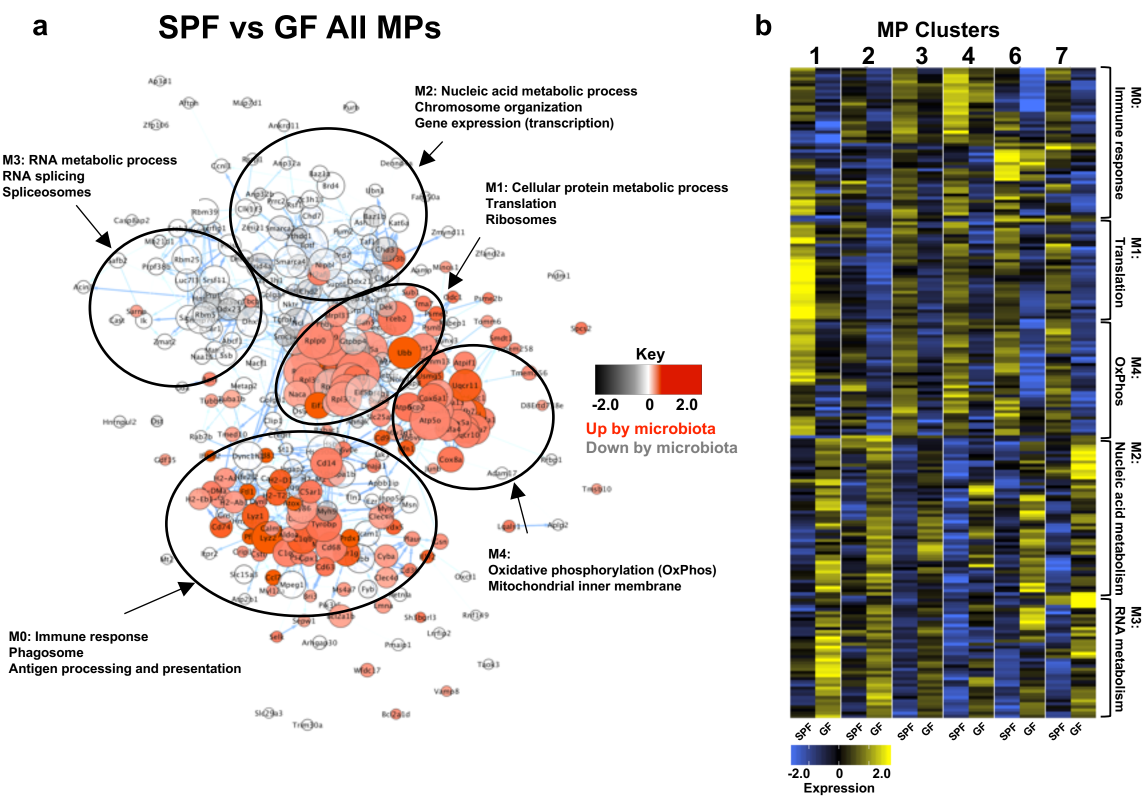

Supplementary Fig. 3

a

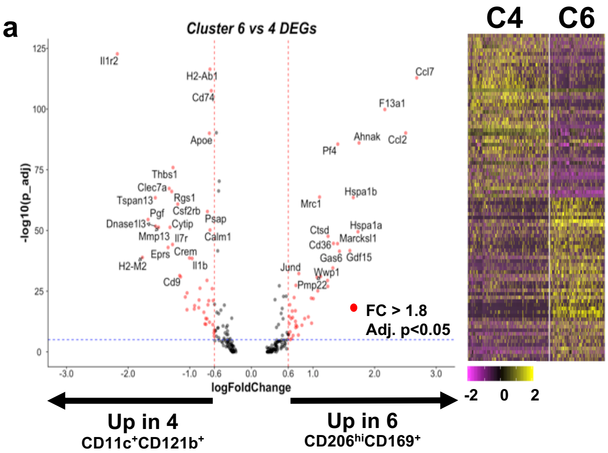

Functional Annotation		
Genes		p.value
	**Up in Cluster 4**	
Slc7a11,Apoe,Psap,Rgs2,Slco3a1,Cyp4f18,Stra6l,Il1b,Thbs1,Eprs,Cd74,Ccr5,Cadm1,Klrb1b,Havcr2,Il7r,Dnase1l3,Apoe,Psap,Slco3a1,Dgat1,Gm2a,H2-M2,H2-Ab1, ...	Organic acid transport	3.38E-08
	Antigen processing and presentation	2.04E-05
	Cell killing	1.23E-06
	Lipid localization	9.09E-05
	Myeloid leukocyte migration	3.26E-06
	**Up in Cluster 6**	
Ccl7,Ccl2,Ccl8,Ccl4,Ccl6,Egr1,Ccl9,Cebpb,F13a1,Gas6,Pf4,Cd36,Hbegf,Igf1,Hmox1,Pdgfa,Plec,Cflar,Stab1,Nrp1,Klf4,Rock2,Tlr2,Icam1,Bag3,P2rx7,Lmna, ...	Cellular response to interleukin-1	3.76E-10
	Wound healing	8.14E-06
	Regulation of vasculature development	5.21E-07
	Apoptotic cell clearance	0.00034
	Myeloid leukocyte cytokine production	0.00018

b

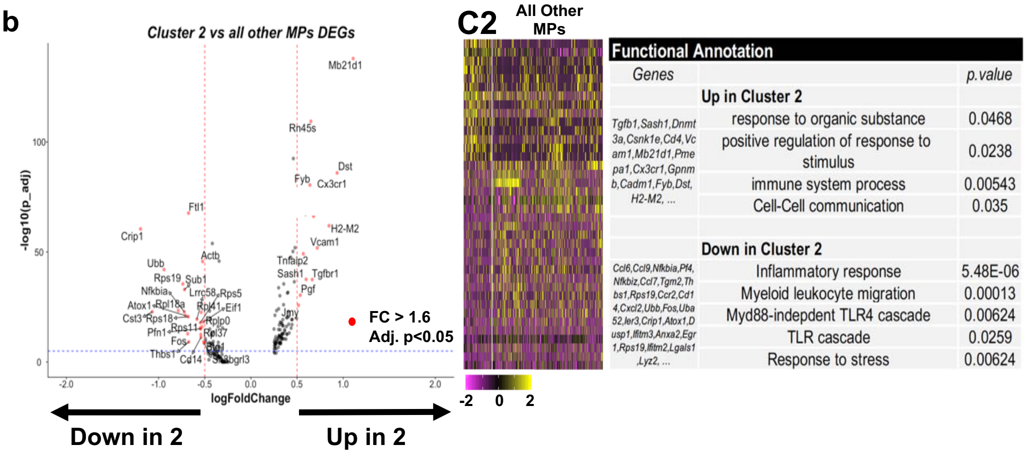

Functional Annotation		
Genes		p.value
	**Up in Cluster 2**	
Tgfbr1,Sash1,Dnmt3a,Csnk1e,Cd4,Vcam1,Mb21d1,Pmepa1,Cx3cr1,Gpnmb,Cadm1,Fyb,Dst,H2-M2, ...	response to organic substance	0.0468
	positive regulation of response to stimulus	0.0238
	immune system process	0.00543
	Cell-Cell communication	0.035
	**Down in Cluster 2**	
Ccl6,Ccl9,Nfkbia,Pf4,Nfkbiz,Ccl7,Tgm2,Thbs1,Rps19,Ccr2,Cd14,Cxcl2,Ubb,Fos,Uba52,Ier3,Crip1,Atox1,Dusp1,Ifitm3,Anxa2,Egr1,Rps19,Ifitm2,Lgals1,Lyz2, ...	Inflammatory response	5.48E-06
	Myeloid leukocyte migration	0.00013
	Myd88-indepdent TLR4 cascade	0.00624
	TLR cascade	0.0259
	Response to stress	0.00624

c

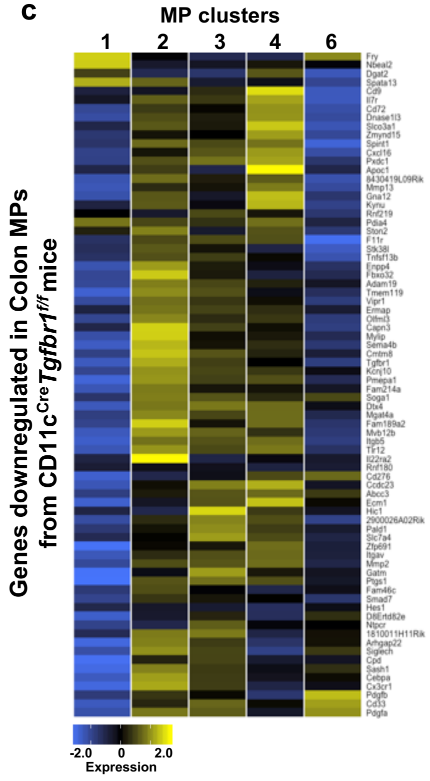

Supplementary Fig. 4

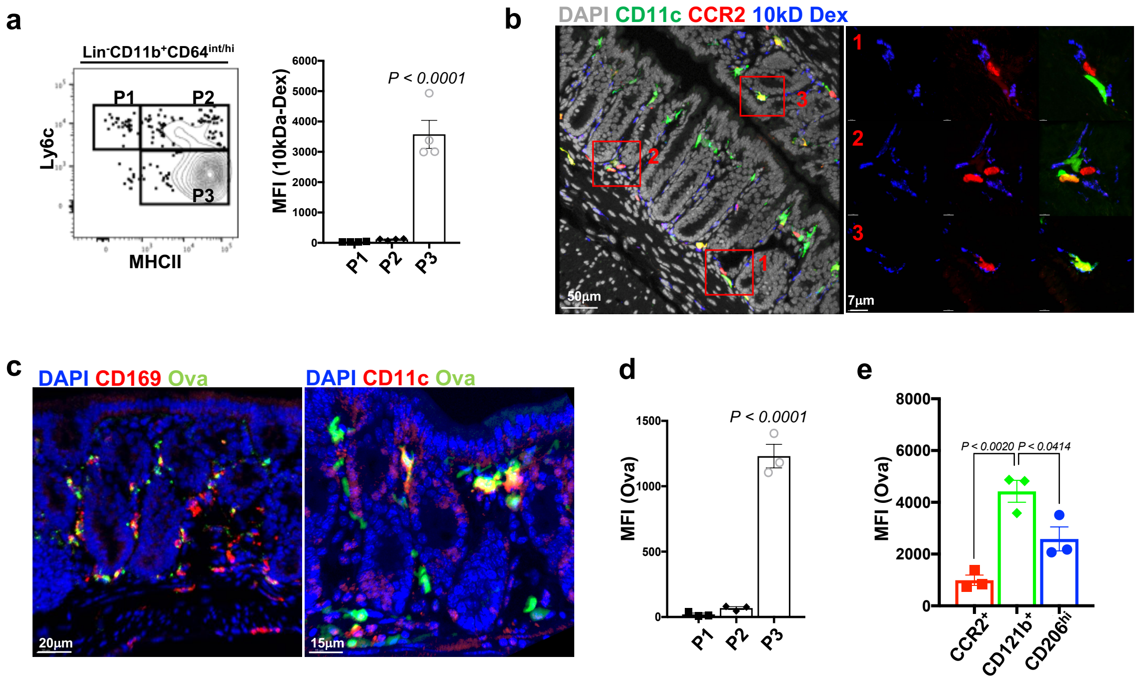

Supplementary Fig. 5

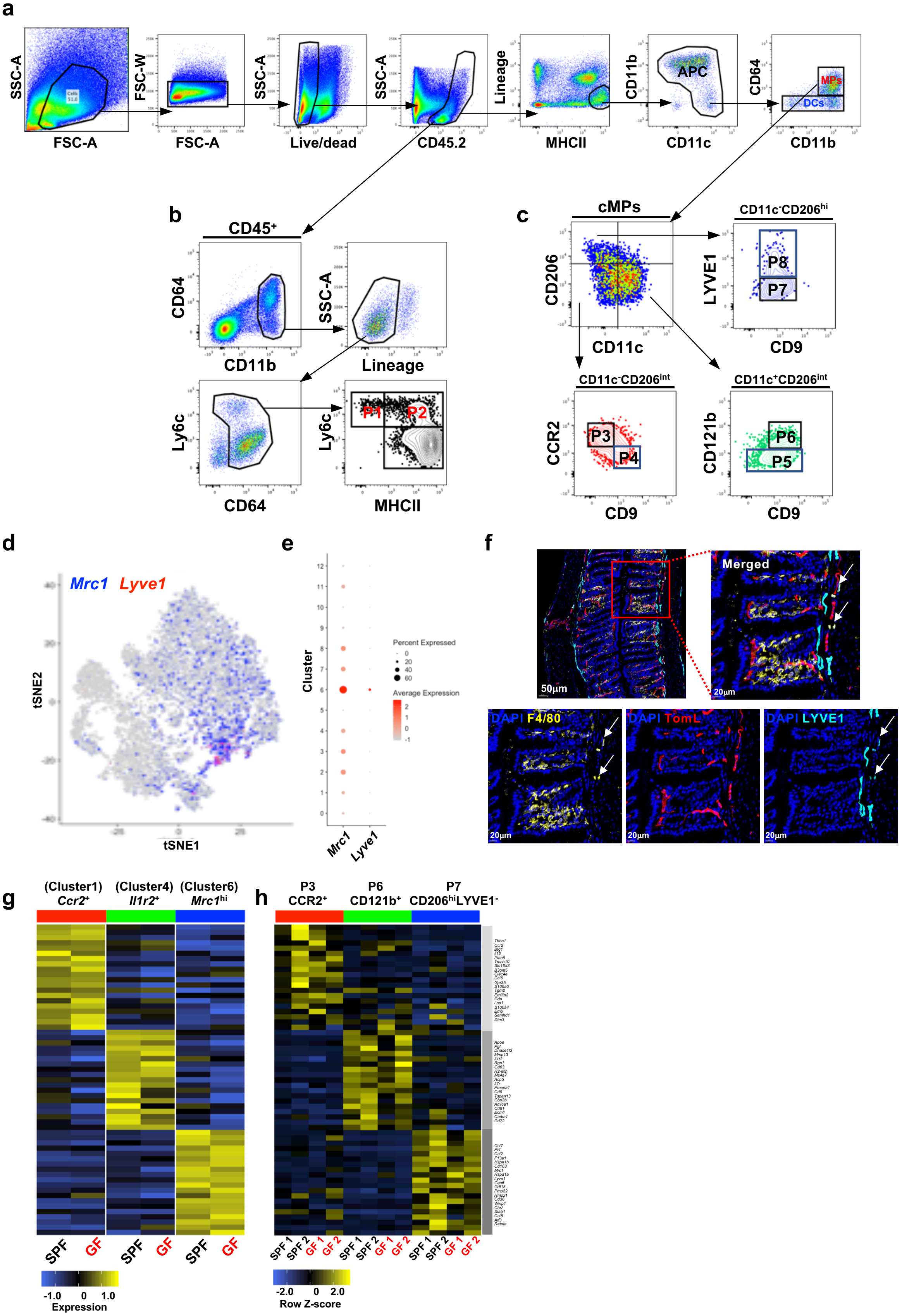

Supplementary Fig. 6

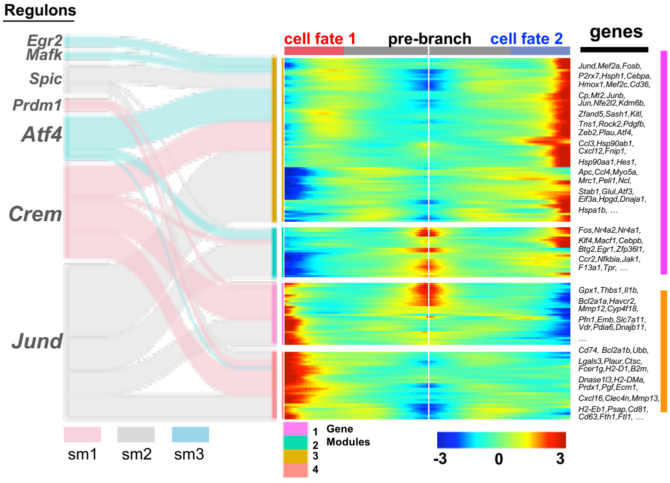

Functional Annotation

ID	Description	GeneRatio	p.adjust
GO:0010035	response to inorganic substance	18/96	4.15E-10
GO:0001667	ameboidal-type cell migration	14/96	1.58E-07
GO:0070997	neuron death	14/96	1.58E-07
GO:1903706	regulation of hemopoiesis	14/96	1.63E-07
GO:0030335	positive regulation of cell migration	14/96	1.62E-06
GO:0032496	response to lipopolysaccharide	13/96	6.67E-07
GO:0001525	angiogenesis	13/96	9.29E-06
GO:0030099	myeloid cell differentiation	11/96	3.36E-05
GO:0050673	epithelial cell proliferation	11/96	4.75E-05
GO:0009611	response to wounding	11/96	0.000118201
GO:0070371	ERK1 and ERK2 cascade	9/96	0.000281276
GO:0043542	endothelial cell migration	8/96	5.21E-05
GO:0042060	wound healing	8/96	0.001353908
GO:0048660	regulation of smooth muscle cell proliferation	7/96	0.00011292
GO:0060326	cell chemotaxis	6/96	0.007534673
GO:1904705	regulation of vascular smooth muscle cell proliferation	5/96	2.67E-05
GO:0009266	response to temperature stimulus	5/96	0.004924852
GO:0007596	blood coagulation	5/96	0.005008143

ID	Description	GeneRatio	p.adjust
GO:2001233	regulation of apoptotic signaling pathway	10/59	1.04E-05
GO:0002697	regulation of immune effector process	9/59	4.05E-05
GO:0002443	leukocyte mediated immunity	9/59	0.000197457
GO:0050900	leukocyte migration	8/59	0.000130017
GO:0051047	positive regulation of secretion	8/59	0.001529001
GO:0019882	antigen processing and presentation	7/59	2.53E-06
GO:0042110	T cell activation	7/59	0.004737192
GO:0030335	positive regulation of cell migration	7/59	0.005111201
GO:0001909	leukocyte mediated cytotoxicity	6/59	4.42E-05
GO:0046942	carboxylic acid transport	6/59	0.002678307

Supplementary Fig. 7

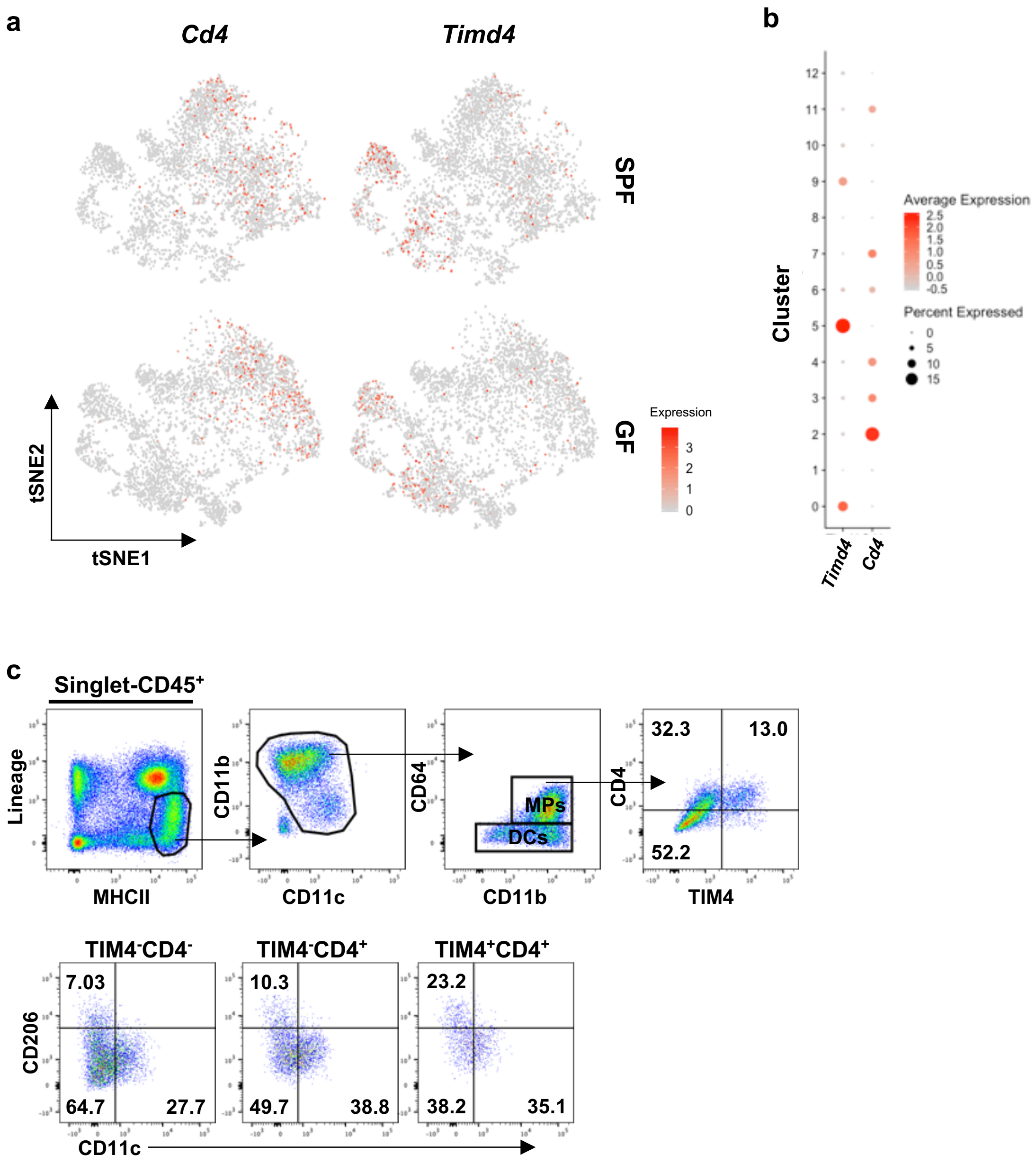

Supplement: Supplementary file 1 — Supplementary Figures [file 41385_2019_228_MOESM1_ESM.pdf]
